# *In silico* prediction of splice-altering single nucleotide variants in the human genome

**DOI:** 10.1093/nar/gku1206

**Published:** 2014-11-21

**Authors:** Xueqiu Jian, Eric Boerwinkle, Xiaoming Liu

**Affiliations:** 1 Division of Epidemiology, Human Genetics and Environmental Sciences, School of Public Health, The University of Texas Health Science Center at Houston, Houston, TX 77030, USA; 2 Human Genetics Center, School of Public Health, The University of Texas Health Science Center at Houston, Houston, TX 77030, USA; 3 Center for Human Genetics, The Brown Foundation Institute of Molecular Medicine for the Prevention of Human Diseases, The University of Texas Health Science Center at Houston, Houston, TX 77030, USA; 4 Human Genome Sequencing Center, Baylor College of Medicine, Houston, TX 77030, USA

## Abstract

*In silico* tools have been developed to predict variants that may have an impact on pre-mRNA splicing. The major limitation of the application of these tools to basic research and clinical practice is the difficulty in interpreting the output. Most tools only predict potential splice sites given a DNA sequence without measuring splicing signal changes caused by a variant. Another limitation is the lack of large-scale evaluation studies of these tools. We compared eight *in silico* tools on 2959 single nucleotide variants within splicing consensus regions (scSNVs) using receiver operating characteristic analysis. The Position Weight Matrix model and MaxEntScan outperformed other methods. Two ensemble learning methods, adaptive boosting and random forests, were used to construct models that take advantage of individual methods. Both models further improved prediction, with outputs of directly interpretable prediction scores. We applied our ensemble scores to scSNVs from the Catalogue of Somatic Mutations in Cancer database. Analysis showed that predicted splice-altering scSNVs are enriched in recurrent scSNVs and known cancer genes. We pre-computed our ensemble scores for all potential scSNVs across the human genome, providing a whole genome level resource for identifying splice-altering scSNVs discovered from large-scale sequencing studies.

## INTRODUCTION

Since pre-mRNA splicing was first discovered in the 1970s (1,2), DNA variations that disrupt normal splicing have been linked to human genetic diseases (3,4,5). Unlike non-synonymous mutations within coding regions that directly alter amino acids by changing the codon, splice-altering mutations influence the normal process of removing introns from the pre-mRNA and rejoining the remaining exons. This normal process is regulated by complicated mechanisms that usually result in the production of different proteins by exon skipping, intron retention, use of different 5′ or 3′ splice sites, etc. which is termed alternative splicing (6). Alternative splicing is very common in human genes; it has been estimated to occur in ∼95% of multi-exon genes (7). The ubiquitous alternative splicing, the previous focus of the scientific community on exonic variants that directly modify protein sequences, and the fact that more than 20% of non-synonymous mutations reported in the Human Gene Mutation Database (HGMD) (8) may also affect splicing (9,10), create challenges in identifying variants that are causal or modifiers for human diseases due to disruption of splicing. Additionally, widespread high-throughput next-generation sequencing (NGS) technology is rapidly generating a large amount of data, which enables the identification of more variants in a shorter time than ever before. For example, a recent study sequenced the whole genome of 962 individuals and identified a total of more than 25 million genetic variants (11). This not only provides us with opportunities to discover novel causal variants but also makes the prioritization of these newly identified variants more challenging in view of the infeasibility of confirming each variant *in vivo*/*in vitro*.

*In silico* tools can take advantage of sequence information to predict the possible effect of a variant based on specific models on a very large scale. This approach has been used to prioritize millions of variants (i.e. distinguish pathogenic mutations from a large number of background variations) and narrow down the search to a relatively small number of variants for laboratory validation. In particular, *in silico* tools for the prediction of variants affecting splicing have been developed that take into account different aspects of the splicing mechanism, which consist of (i) splicing signals, including the 5′/3′ splice site and the branch point; (ii) splicing regulatory elements, i.e. exonic/intronic splicing enhancers/silencers; and (iii) the spliceosome and other *trans*-acting elements that bind to *cis*-acting elements. Some tools have had successful application in predicting splicing defects. For instance, MaxEntScan, a tool that predicts the 5′/3′ splice site (12), correctly predicted three apparently nonsense, missense or silent mutations as disrupting normal splice sites in the *ATM* gene that are responsible for ataxia-telangiectasia (13). ESEfinder, a tool that predicts exonic splicing enhancers (ESEs) (14), has successfully predicted the loss of a putative ESE motif in the *SMN2* gene as the cause of spinal muscular atrophy (15). These examples imply that *in silico* tools have the potential to be utilized for prioritization of variants that may disrupt splicing in the NGS era.

Recently, we reviewed some of the most commonly used *in silico* tools for splicing defect prediction and noted that the major problem that prohibits the use of these tools in research and clinical practice is the difficulty in interpreting the output (16). One reason for this difficulty is that there is no unified standard to measure how splicing signals change when one allele is substituted by another because most tools only output prediction scores for potential splice sites given an input DNA sequence. Another reason is the lack of large-scale studies to evaluate the predictive performance of these tools. To make a more thorough comparison of existing prediction tools and provide a directly interpretable score for splice-altering variants, we evaluated eight *in silico* tools and constructed prediction models using ensemble learning methods that take advantage of some of these tools in order to further improve prediction. Because the relatively conserved sequences at the two ends of an intron (splicing consensus sequences) are the prominent *cis*-acting elements of splicing (17) that have been the focus of most existing tools, and single nucleotide variants (SNVs) are the most commonly observed variants in the human genome (18), the present study is restricted to SNVs within splicing consensus regions (−3 to +8 at the 5′ splice site and −12 to +2 at the 3′ splice site) defined by Burge *et al*. (17); we named these variants scSNVs. We validated our models, pre-computed our ensemble prediction scores for all potential scSNVs across the human genome and applied them to scSNVs from the Catalogue of Somatic Mutations in Cancer (COSMIC) database (19).

## MATERIALS AND METHODS

### Data sources

Positive (i.e. splice-altering) variants were downloaded from three databases: (i) the HGMD Professional Version 2013.1, which contains more than 13 000 mutations with consequences for mRNA splicing (8); most (more than 8000) are located at invariant GT-AG sites (the first two and last two sites of an intron), while the remaining sites are mostly exonic; (ii) the SpliceDisease database, which collects and curates experimentally supported data of RNA splicing mutations and disease (20); this database integrated 2337 splicing mutation-disease entries, including 303 genes and 370 human diseases from 898 publications; and (iii) the Database for Aberrant Splice Sites (DBASS), which contains 577 and 307 records of mutation-induced and disease-causing aberrant 5′ and 3′ splice sites, respectively (21). Negative variants were retrieved from the 1000 Genomes Project phase 1 data (18), which contain genomes of 1092 individuals from 14 populations that can be used as controls for comparisons with other samples. To further validate our final models, an additional independent test set was retrieved from the published data of Houdayer *et al*., in which the impact of the variants was experimentally validated (22).

### Splicing variant filtering strategy

The following strategies were used when recruiting variants from the databases into our positive group: (i) based on the National Center for Biotechnology Information Reference Sequence (RefSeq) database release 59 (23), we only included variants within the splicing consensus regions (−3 to +8 at the 5′ splice site and −12 to +2 at the 3′ splice site) at the exon/intron boundaries of protein-coding genes; (ii) within the consensus regions, all variants at GT-AG sites were excluded, because these sites are so invariant that almost all mutations that occur at these sites affect splicing and most tools can predict their impact with very high accuracy (22,24); (iii) only single nucleotide substitutions (i.e. SNVs) were retained; (iv) variants were excluded if information provided by the database did not contain biological evidence (e.g. merely computational predictions or statistical associations); and (v) to avoid duplication, variants present in more than one database were only counted once. The first three criteria were also applied to the recruitment of negative variants from the 1000 Genomes Project phase 1 data. Furthermore, additional filtering strategies were implemented: we chose variants within genes that have only one annotated transcript in RefSeq database release 59 (this only applies to recruitment of negative variants) (23), and we only chose variants with minor allele frequency >0.05 in combined populations of European ancestry. The rationale is that as individuals of European ancestry are the most commonly studied subjects; if a common variant alters splicing, it is highly likely the alternatively spliced transcript has been reported in this population. In contrast, a common variant in a gene without alternative transcripts reported is unlikely to alter splicing of that gene. For the additional test set, we chose the variants reported in the work of Houdayer *et al*. that are (i) within splicing consensus regions defined above; (ii) single nucleotide substitutions; and (iii) not in our dataset (22). All variants were annotated using ANNOVAR, a software package that performs functional annotation of genetic variants from high-throughput sequencing data (25) and based on human reference sequence assembly GRCh37/hg19.

### Choice of*in silico* prediction tools

A total of eight tools were examined because they provide command line interface and can conduct ‘batch’ analysis, which is easier, quicker and more convenient for the submission of a large number of variants. Among them, the Position Weight Matrix (PWM) model (26), MaxEntScan (MES) (12), Splice Site Prediction by Neural Network (NNSplice) (27), GeneSplicer (28) and Human Splicing Finder (HSF) (29) are integrated into a commercial annotation software package called Alamut (Interactive Biosoftware, Rouen, France). We used its high-throughput version, Alamut Batch, to obtain prediction scores of these five tools simultaneously. Because NetGene2 (30), GENSCAN (31) and SplicePredictor (32) provide their source codes, we obtained their outputs separately (Supplementary Table S1). All tools provide one prediction score for the wild-type allele and one for the mutant allele of a SNV.

### Score missing rate and score transformation

For each scSNV, we first obtained the prediction scores for its wild-type and mutant alleles using all eight tools (Supplementary Table S2). For negative variants, we considered major alleles as wild-type and minor alleles as mutant. To correctly predict the consequence of a scSNV, a tool should be able to identify the wild-type splice site first. Because the wild-type allele represents the true splice site and a higher score implies a higher probability of being a true splice site, the wild-type score for a variant should not be zero; otherwise, the tool is not able to detect the true splice site. Therefore, we first carried out a screening step with all eight tools by examining their ability to predict the true splice site for all variants in our dataset, and a score was considered missing if the score for the wild-type of a variant was zero. We excluded tools with missing score rates >5% from our evaluation analysis. To compare the predictive performance of different tools, we transformed two scores for each variant to a single score that measures the scale of change caused by the variant via calculation of the score difference for each variant. The larger the difference is, the more likely the variant alters splicing. For a given tool, if the score had a range (e.g. 0 to 100 for PWM), we calculated two score variations, one that was relative (score difference divided by the wild-type score) and one that was absolute (score difference divided the by score range). If the score did not have a finite range (e.g. MES score is a log-odds ratio), only a relative score variation was calculated. For each tool, score variations were not calculated for variants whose wild-type score was zero (i.e. missing). These variants were discarded from the evaluation analysis for this individual tool.

### ROC analysis and model construction using ensemble learning methods

We performed receiver operating characteristic (ROC) analysis (33) with 10-fold cross-validation on the seven score variations. We defined the cutoff value as the score variation above which the results should be considered positive. We calculated sensitivity and specificity across the whole spectrum of possible cutoff values by comparing the predictive results with the ‘true’ results for a series of cutoff values from the minimum to the maximum and plotted all pairs to form a ROC curve. For each score variation, we plotted the ROC curve, obtained areas under the ROC curve (AUC), and identified the optimum cutoff value that maximized accuracy in the cross-validation training set to calculate the accuracy, sensitivity, specificity, positive predictive value (PPV) and negative predictive value (NPV) in the corresponding test set. All these measures were averaged from 10 cross-validation runs. We also compared AUCs of each pair of score variations using 10-fold cross-validated paired *t*-test (34). Considering that mechanisms for the identification of 5′ and 3′ splicing signals may differ, we also divided our dataset into variants within 5′ or 3′ splicing consensus regions and performed the analysis separately to see if a tool performed better for specific regions. ROC analysis was performed using R version 3.1.0 (R Foundation for Statistical Computing, Vienna, Austria). All ROC curves were drawn using the R package ROCR (35).

Two ensemble learning methods, adaptive boosting (AdaBoost) (36) and random forests (37), were used to construct new models that improve the prediction. The two R packages used were ada (38) and randomForest (39), respectively. Training on our dataset using both ensemble learning methods, we inputted different combinations of parameter values to identify those that maximize the AUC. The following settings were chosen because they performed best for their respective conditions. For AdaBoost, we implemented the ‘gentle boost’ algorithm under the ‘logistic loss’ function while keeping other arguments at the default. For random forests, we used all default settings. We conducted ROC analysis as described above to evaluate the new models. To further improve the predictive performance, two conservation scores (phyloP46way_placental and phyloP46way_primate (40)) and two whole-genome functional prediction scores (CADD_raw and CADD_phred (41)) were added to both models because they had a score missing rate <0.05 for all sites within splicing consensus regions on each chromosome (data not shown). These scores were obtained separately (Supplementary Table S1). When training new models with 11 scores, we ran the same process except for replacing the ‘gentle boost’ algorithm with the ‘real boost’ algorithm in AdaBoost. For all ensemble methods, we also assessed the relative importance of each individual score, which was measured by the frequency of a score selected for boosting for AdaBoost and the mean decrease in accuracy for random forests.

### Validation and application

After the best models were identified, they were retrained using all variants in our dataset. To further validate our models, we obtained our ensemble scores for all variants in the additional test set and used the optimum cutoff value identified in the ROC analysis (0.6) to calculate the sensitivity, specificity and accuracy. We also used the retrained models to compute ensemble prediction scores for all potential scSNVs across the human genome annotated by either RefSeq database release 62 (23) or Ensembl database release 73 (42).

We applied our scores to the COSMIC dataset (19), which curates comprehensive information on somatic mutations in human cancer. As of its v68 release, 1 627 878 mutations were documented from 981 720 tumor samples within 25 660 genes, of which 522 genes are known to be involved in cancer promotion (Cancer Gene Census) (43). We first identified all scSNVs in COSMIC and obtained our ensemble prediction scores. Then, we tested whether predicted splice-altering scSNVs were enriched in recurrent scSNVs (measured by the number of times a variant was observed) and in known cancer genes using the Chi-squared test for trend in proportions and Pearson's Chi-squared test, respectively.

## RESULTS

### Dataset, *in silico* tools and missing score rates

After filtering data according to our inclusion and exclusion criteria, 1164 unique splice-altering scSNVs within 408 genes from three databases constituted our positive group, among which 790 were from HGMD (8), 266 from the SpliceDisease database (20) and 108 from DBASS (21). Our negative group consisted of 1795 scSNVs within 1447 genes from the 1000 Genomes Project phase 1 data (18). Our total sample size was 2959 (within 1804 genes), of which 1682 were within the 5′ splicing consensus region (positions −3 to +8) and 1277 were within the 3′ splicing consensus region (positions −12 to +2) (17) and 1881 were intronic and 1078 were exonic (Table 1). All variants were within the splicing consensus regions, but outside of the GT-AG sites. To see the complete list of these 2959 positive and negative scSNVs, please refer to Supplementary Table S2. To compare the predictive performance of PWM, MES, NNSplice, GeneSplicer, HSF, NetGene2, GENSCAN and SplicePredictor, we first examined the ability of these tools to identify wild-type splice sites for all variants in our dataset and treated them as a missing score if the wild-type score of a variant was zero. Due to extremely high missing score rates (Table 2), we excluded GeneSplicer, GENSCAN, NetGene2 and SplicePredictor from further evaluation and limited our comparison to PWM, MES, NNSplice and HSF. Because MES score does not have a finite range (log-odds ratio), we only calculated one score variation for MES (relative). Each of the remaining three tools had two score variations (relative and absolute). Therefore, seven score variations were entered into our ROC analysis.

**Table 1.** Summary of the dataset used in the present study

Data source	5′	3′	Intronic	Exonic	Total
Positive					
HGMD	725	65	2	788	790
SpliceDisease	182	84	235	31	266
DBASS	63	45	96	12	108
Subtotal	970	194	333	831	1164
Negative					
1000 Genomes	712	1083	1548	247	1795
Total	1682	1277	1881	1078	2959

**Table 2.** Missing rates of the prediction scores for eight *in silico* tools

Tool	No. of missing	Missing rate
PWM	77	0.026
MES	82	0.028
NNSplice	68	0.023
HSF	66	0.022
GeneSplicer	563	0.190
GENSCAN	2466	0.833
NetGene2	1887	0.638
SplicePredictor	2252	0.761

### PWM and MES have the best predictive performance among individual tools

**Figure 1.**
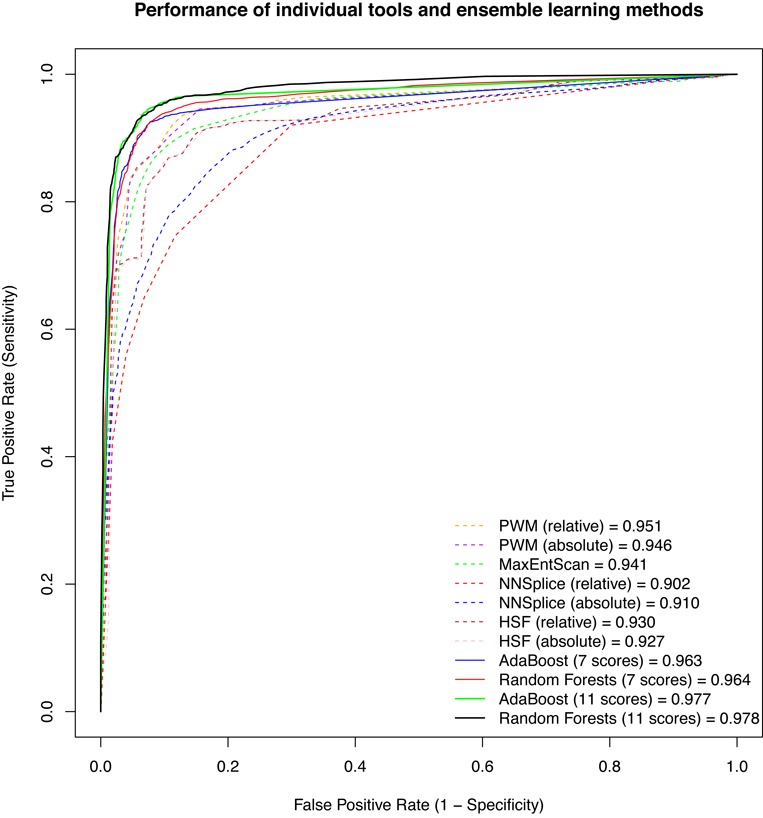
Averaged ROC curves for seven individual scores and four ensemble scores with 10-fold cross-validation.

**Figure 2.**
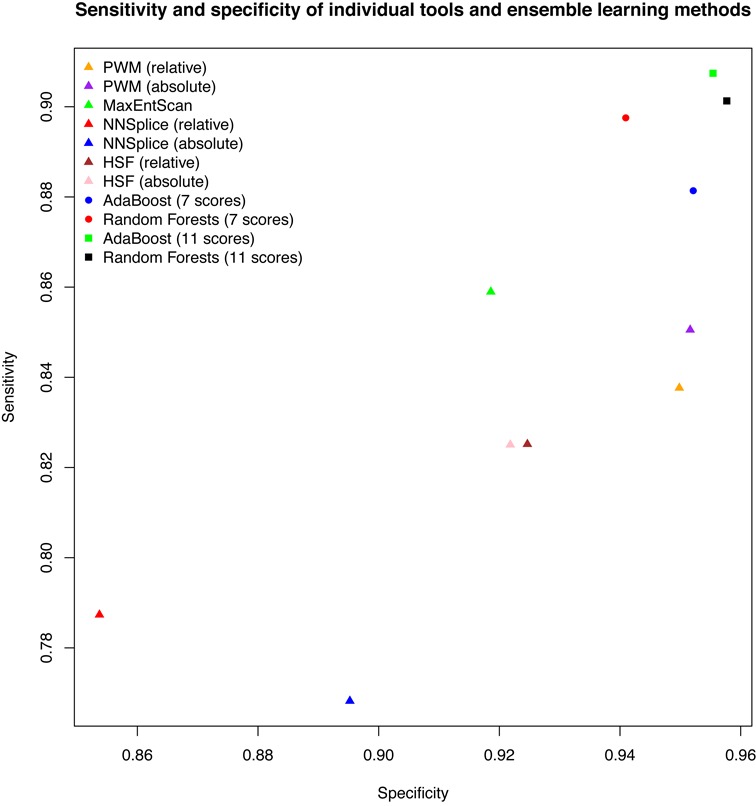
Plots of sensitivity and specificity for seven individual scores and four ensemble scores using cutoff values that maximize accuracy. All measures are reported as averages based on 10-fold cross-validation.

**Figure 3.**
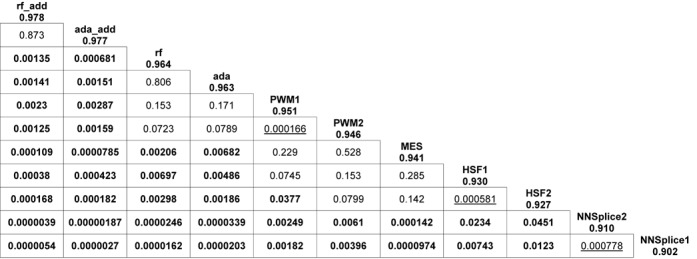
*P*-values of 10-fold cross-validated paired *t*-test for AUCs between any two scores. The scores are ordered by AUC. The further the distance between a cell and its vertical/horizontal labels, the larger the difference between the AUCs for vertical/horizontal scores. Score pairs with significantly different AUCs are **bold**. Comparison between different score variations of the same tool are underlined. The remaining represent the non-significant AUC differences.

ROC curves are illustrated in Figure 1. For each score variation, 10 curves from the cross-validation runs were averaged to one curve. The 10 AUCs were also averaged. Although all score variations were maintained at a high level of overall performance (all AUCs > 0.9), PWM outperformed all other tested methods, with AUCs of 0.951 (relative) and 0.946 (absolute). MES had a similar performance, with an AUC of 0.941. HSF performed a little poorer worse, with AUCs of 0.930 (relative) and 0.927 (absolute), followed by NNSplice with AUCs of 0.902 (relative) and 0.910 (absolute). To determine the qualitative predictive performance for each score variation, the mean sensitivity and specificity from 10 cross-validation runs were plotted (Figure 2). For example, when the cutoff value for PWM (absolute) was set to 0.06 (which maximized accuracy in the cross-validation training set), the accuracy was 0.911, with a corresponding sensitivity of 0.851 and specificity of 0.952 in the cross-validation test set. The full list of these evaluation measures for all score variations as well as their PPVs and NPVs are summarized in Table 3. The *P*-values of the 10-fold cross-validated paired *t*-test (34) for the difference in AUCs between any pair of score variations are illustrated in Figure 3. The results for stratified analyses are consistent with those for pooled analyses in that PWM and MES performed best, but no improvements in AUC were observed by predictions for any tool at either the 5′ or 3′ splice site (results are similar or weaker and are not shown).

**Table 3.** Mean values of the evaluation measures for the 11 scores based on 10-fold cross-validation

Score	AUC	Cutoff	Accuracy	Sensitivity	Specificity	PPV^a^	NPV^b^
PWM1	0.951	0.075	0.905	0.838	0.950	0.918	0.897
PWM2	0.946	0.062	0.911	0.851	0.952	0.922	0.904
MES	0.941	0.217	0.895	0.859	0.919	0.875	0.908
NNSplice1	0.902	0.146	0.827	0.787	0.854	0.782	0.860
NNSplice2	0.910	0.125	0.845	0.768	0.895	0.830	0.855
HSF1	0.930	0.044	0.885	0.825	0.925	0.879	0.889
HSF2	0.927	0.039	0.883	0.825	0.922	0.875	0.889
AdaBoost	0.963	0.708	0.924	0.881	0.952	0.923	0.925
Random forests	0.964	0.515	0.923	0.898	0.941	0.911	0.932
ada_add^c^	0.977	0.612	0.937	0.907	0.955	0.930	0.941
rf_add^d^	0.978	0.598	0.935	0.901	0.958	0.935	0.936

^a^PPV: positive predictive value.

^b^NPV: negative predictive value.

^c^ada_add: AdaBoost with four additional scores included.

^d^rf_add: random forests with four additional scores included.

### Models constructed using ensemble learning methods further improve predictions and are validated on an additional test set

Using two ensemble learning methods, AdaBoost (36) and random forests (37), we constructed new models that take advantage of all seven score variations. Ensemble learning methods have a better overall performance than any individual method, with similar AUCs (AdaBoost: 0.963; random forests: 0.964) (Figure 1, Table 3). To further improve the predictive performance, two conservation scores (phyloP46way_placental and phyloP46way_primate (40)) and two whole-genome functional prediction scores (CADD_raw and CADD_phred (41)) were added to both models. Addition of these four scores further improved both models, with AUCs of 0.977 for AdaBoost and 0.978 for random forests (Figure 1, Table 3), with optimum sensitivity >0.9 and specificity >0.95, which were significant increases compared with any other method evaluated (Figure 2, Table 3). The *P*-values of the 10-fold cross-validated paired *t*-test for the difference in AUCs between ensemble methods and individual methods are illustrated in Figure 3. Relative importance for each individual score is summarized in Supplementary Table S3. All ensemble methods outputted a probability score for each variant that reflects the confidence that the variant alters splicing.

We then validated these two models (retrained using all data instead of cross-validation training data) on an additional test set curated from a published study (22). A total of 65 variants were located within splicing consensus regions (32 within *BRCA1* and 33 within *BRCA2*), 56 of which were SNVs. Due to annotation discrepancies, we obtained our prediction scores for 54 scSNVs; nine of these were also in our dataset. Therefore, the additional test set consisted of 45 scSNVs with experimentally validated splicing outcomes (Supplementary Table S4). The original publication classified these variants into three classes: 1S (no effect on splicing), 2S (partial effect or effect on alternative splicing) and 3S (severe effect on splicing) (22). There were only two scSNVs in the 2S class that slightly changed alternative splicing; because this was not the goal of our prediction, we considered them negative. Consequently, 19 scSNVs were positive and 26 were negative. Using the optimum cutoff point of 0.6 from our evaluation analysis, all 19 positive scSNVs were correctly predicted by AdaBoost, with six false positives, yielding a sensitivity of 1 and specificity of 0.77 (accuracy = 0.87). For random forests, there was one missing score for a negative scSNV. All 19 positive scSNVs were correctly predicted, and there were only three false positives, yielding a sensitivity of 1 and specificity of 0.88 (accuracy = 0.93).

### Pre-computed ensemble prediction scores for all potential scSNVs across the human genome

Using our two retrained models, we pre-computed our ensemble scores for all potential scSNVs across the human genome (named ‘ada_score’ and ‘rf_score’ for prediction scores computed using AdaBoost and random forests, respectively). Based on RefSeq database release 62 (23) and Ensembl database release 73 (42), we curated a total of 15 679 428 potential scSNVs; 15 030 435 had prediction scores and the remaining 4.14% were missing due to annotation discrepancies. Because the R package ada (used to train the AdaBoost model) (38) can handle missing values whereas randomForest (used to train the random forests model) (39) cannot, all 15 030 435 scSNVs had an ada_score, whereas 214 616 scSNVs with missing individual scores did not have an rf_score (missing rate 1.43%). These statistics, as well as the missing rates of the four splicing prediction scores, are tabulated in Supplementary Table S5.

### Splice-altering scSNVs are enriched in recurrent scSNVs and in known cancer genes in COSMIC

To illustrate the application potential of our prediction models, we investigated the distribution of predicted splice-altering scSNVs extracted from the COSMIC database (19). We obtained our ensemble scores for all scSNVs in COSMIC and considered those with either score >0.6 as splice-altering. The number of scSNVs and the number of predicted splice-altering scSNVs that were observed once or twice, three or four times and five or more times in COSMIC are summarized in Table 4. It is obvious that as the number of times scSNVs were observed increases (i.e. the more likely they were driver mutations), the proportion of predicted splice-altering scSNVs increases. The Chi-squared test for trend in proportions showed that this trend is significant (*P* = 0.0004114). We also divided these scSNVs into two groups: one within known cancer genes (Cancer Gene Census) (43) and the other within non-cancer genes. The number of scSNVs and the number of predicted splice-altering scSNVs within cancer genes or non-cancer genes are summarized in Table 5. Approximately 79% of scSNVs within cancer genes were predicted to be splice-altering, versus 70% within non-cancer genes. Pearson's Chi-squared test showed that this difference is highly significant (*P* < 2.2e − 16). Among the 2025 predicted splice-altering scSNVs within cancer genes (Supplementary Table S6), 58 are synonymous and 83 are intronic. Because synonymous and intronic SNVs do not to change amino acids and therefore might be neglected by most functional prediction tools, further studies are needed to investigate the validity of their probable impact on splicing and their roles in cancer pathogenesis.

**Table 4.** Trend for the distribution of predicted splice-altering scSNVs in COSMIC

No. of times observed	No. of scSNVs	No. of predicted splice-altering scSNVs	Proportion
1 or 2	41 455	29 322	0.707
3 or 4	299	222	0.742
5 or more	129	109	0.845

**Table 5.** Distribution of predicted splice-altering scSNVs within cancer/non-cancer genes in COSMIC

Gene	No. of scSNVs	No. of predicted splice-altering scSNVs	Proportion
Cancer	2566	2025	0.789
Non-cancer	39 317	27 628	0.703

## DISCUSSION

Recognizing the interpretation gap of *in silico* methods for predicting splice-altering variants, the present study aimed to (i) compare the predictive performance of some of the currently available *in silico* tools; (ii) construct prediction models that take advantage of multiple tools and provide directly interpretable scores; and (iii) provide a whole genome level resource for identifying splice-altering scSNVs discovered from large-scale sequencing studies.

### Predictive performance of individual tools and models constructed using ensemble learning methods

Our ROC analysis showed that PWM and MES had the largest AUCs (Figure 1, Table 3), which is in accordance with the results of two previous studies (22,24). The two models were initially proposed based on slightly different definitions of splicing consensus sequences. PWM was trained based on sequences from −3 to +6 at the 5′ splice site and from −14 to +1 at the 3′ splice site (26); MES was trained based on sequences from −3 to +6 at the 5′ splice site and from −20 to +3 at the 3′ splice site (12). The sequences used to train both methods have a large overlap with the consensus sequences used in the present study (−3 to +8 at the 5′ splice site and −12 to +2 at the 3′ splice site). Therefore, it is not surprising to see that they performed better than other methods. Although all the tools we compared were developed for prediction of both 5′ and 3′ splice sites, we explored the possibility that tools may perform differently for these two sites and better than the overall prediction. Unfortunately, we observed similar or weaker performance compared with the pooled analysis. One possible reason is that these methods indeed perform equally well (or poorly) for both 5′ and 3′ splice sites. Another possible explanation is that we simply did not have a sufficiently large sample size to observe differences (especially for the 3′ splice site because there are relatively few cases compared with controls in our dataset). Although the predictive performances of PWM and MES were already at a high level, our models still significantly improved predictions by more than 2% in terms of AUC values (Figures 1 and 3, Table 3), indicating that ensemble learning methods have the ability to take advantage of different aspects of splicing implemented by different individual tools. The two ensemble learning methods showed different patterns in the relative importance of each individual score (Supplementary Table S3). The frequencies of all individual scores for both AdaBoost models were ∼0.1, indicating that all scores contributed almost equally. By contrast, MES was more important than other tools for the model constructed using random forests without additional scores. When adding the four additional scores, phyloP46way_placental shared the most important individual scores with MES. The differences in relative importance in the two ensemble learning methods reflect their distinct algorithms used for training.

To further validate our two best models, we made use of experimentally validated scSNVs from a previous study (22). Both models achieved 100% sensitivity. Random forests had higher specificity than AdaBoost (0.88 versus 0.77), but it might suffered from missing scores. To maximize favorable factors while minimizing unfavorable ones for both scores, we took into account both scores when predicting a variant. Conservatively, we considered a variant to be positive (i.e. splice-altering) only if both scores exceeded the optimum cutoff value of 0.6. This resulted in only three false positives, yielding 100% sensitivity and 88.5% specificity (accuracy = 93.3%). Validation of our models on an independent test set further confirmed their power in predicting splice-altering scSNVs.

### Application of ensemble prediction scores to scSNVs in the human genome and in COSMIC

Both ensemble scores computed using AdaBoost and random forests are the probabilities of a variant being splice-altering. Note that as a probability, the score is not a reflection of the effect size (e.g. how damaging the variant is), but rather the confidence that it alters splicing. When applying our models to the human genome and data from COSMIC, we defined a variant to be splice-altering if either its ada_score or rf_score was larger than 0.6, the optimum cutoff point identified in our evaluation analysis. For exploratory purposes, one can choose a lower cutoff point (e.g. 0.5) to increase sensitivity at the expense of specificity.

One goal of cancer genetics is to distinguish driver mutations from many background mutations that are causal for the cancer. Recurrent mutations in cancer are considered more likely to be driver mutations; thus, detecting recurrent mutations in cancer is an important way to identify cancer driver mutations. Because it has been well documented that splice-altering mutations can contribute to cancer pathogenesis (44), it is natural to infer that recurrent scSNVs in cancer can be thought of as candidate cancer driver mutations by affecting normal splicing (45,46). The COSMIC database provides the frequency with which a variant was observed in cancer sequencing studies. Thus, if our models performed well, we expected that predicted splice-altering scSNVs should be enriched in recurrent scSNVs. The proportions of predicted splice-altering scSNVs clearly demonstrated a statistically significant trend (Table 4). Cancer genes were identified because their mutations had been causally implicated in cancer (43). Another inference we made is that the proportion of splice-altering scSNVs should be larger in cancer genes than in non-cancer genes. We tested whether our models could correctly predict this difference, and the result was highly significant (Table 5).

Synonymous mutations have long been considered functionally irrelevant to diseases because they do not alter amino acids. As a result, most functional prediction tools take little account of such mutations. However, recent analysis by Supek *et al*. showed that synonymous mutations can be oncogenic by affecting splicing (e.g. recurrent synonymous mutations in the *TP53* gene can inactivate adjacent splice sites) (47). This emphasized the importance of synonymous mutations, especially those in the vicinity of splice sites, in altering mRNA splicing in cancer. We identified 58 synonymous scSNVs from cancer genes that are predicted to be splice-altering using our prediction models (Supplementary Table S6). These may be used as candidates for further validation. Interestingly, one synonymous scSNV in the *TP53* gene (a C to T single nucleotide substitution at position 7576853 on chromosome 17) was predicted to be splice-altering with high confidence (both ensemble scores are nearly 1), which is in accordance with the finding of Supek *et al.*

### Strengths

To the best of our knowledge, the current study is by far the largest evaluation analysis of *in silico* tools for splicing defect prediction. The largest previous study that was conducted by Desmet *et al*., which only included 623 variants (24) and was subject to more random errors. We collected almost 3000 variants so that the power of our study was significantly increased. The variants in our analysis span more than 1800 genes, resulting in a more universal analysis. Consequently, our comparison and models based on these variants should not be gene specific and should be generalizable to the whole genome. Prior to ROC analysis, we created a criterion for determining the missing rate of prediction scores to pre-screen tools to ensure that those that entered our evaluation analysis were at least able to predict the true splice site. Using ROC analysis with cross-validation, we evaluated both the quantitative (measured by AUC) and the qualitative (measured by accuracy, sensitivity, specificity, etc.) predictions of these tools, which provided a complete picture of their performance. Our two best models were constructed using AdaBoost and random forest and significantly outperformed all other individual tools we evaluated in both quantitative and qualitative predictions. This gave us the opportunity to calculate a directly interpretable score. As a probability, our ensemble prediction score is straightforward to interpret: it is a ‘per substitution’ instead of a ‘per nucleotide’ score (which existing tools output), and the value of the score is a probability so that one can directly determine the likelihood a variant is splice-altering. We validated our scores on an independent test set and the COSMIC dataset. Both results indicated that the predictions are satisfactory. A recently developed new tool, MutPred Splice (48), outputs a probability score similar to that described for the present study, but only for exonic variants and its performance on our dataset (exonic subset) was not satisfactory (AUC = 0.715, data not shown). Furthermore, we pre-computed our ensemble scores for all potential scSNVs across the human genome, which should significantly facilitate their use in future research, especially for large-scale sequencing-based studies. We have incorporated the two scores as well as their corresponding annotation information as an attached database called dbscSNV into dbNSFP. dbNSFP is a database for functional prediction and annotation of non-synonymous and splice-altering SNVs (49,50). Both dbscSNV and dbNSFP are freely downloadable at https://sites.google.com/site/jpopgen/dbNSFP.

### Limitations and future directions

Despite the advantages discussed above, several limitations of the present study merit discussion. Because we attempted to recruit as many deleterious splice-altering variants as possible, it was infeasible to validate every single one in a wet lab. To minimize misclassification, we implemented stringent quality control during the filtration step. However, it was still likely that a small proportion of variants were false positives. As the scientific literature grows, there will be an increase in confirmed pathogenic splice-altering variants available for use. Another limitation is that controlling for potential confounding effects of alternative splicing on *in silico* predictions remains a major problem for selecting negative variants because alternative splicing is so wide spread (7). Although we have chosen variants from well-studied populations and restricted our analysis to genes with only one annotated transcript, there are still many more examples of which we are unaware. A possible solution is to take advantage of the ability of whole transcriptome shotgun sequencing (RNA-seq) to detect alternative splicing (51,52) to recruit variants that do not cause any alternative splicing. Several attempts have been made to integrate RNA-seq data into *in silico* tools in order to improve predictions (e.g. SpliceFinder (53) and Veridical (54)). Although their reliability and ease of use require further evaluation, integration of multi-information into one single tool has become a trend.

Our dataset was limited to SNVs because they are the most common type of genetic variants observed. We also restricted our analysis to variants within splicing consensus regions, considering that variants that are close to the splice sites are more likely to affect splicing. Future work may extend to deeper intronic/exonic variants, as well as other types of variants such as insertions and deletions. In the present study, we did not distinguish different outcomes of splice-altering variants (e.g. exon skipping, intron retention and cryptic splice site) due to lack of information. As a result, we can only provide an overall prediction for a variant (splice-altering or not). Future efforts should be made for more specific outcomes to make the prediction more informative. We compared eight *in silico* tools with the primary goal of predicting 5′/3′ splice sites. Other *cis*-acting elements of splicing, such as branch points and ESEs, were not considered. Because a splicing event is the result of the interaction between many *cis*-acting and *trans*-acting splicing elements and a single tool cannot take all these elements into account, these tools can only be used as a means of screening and filtration, and wet lab validation will always be the gold standard (55,56). A possible way to improve predictions is to include prediction algorithms that take into account other aspects of splicing (e.g. LaSSO (57) for branch point prediction and RESCUE-ESE (58) and ESEfinder (14) for ESE prediction).

An implicit assumption when interpreting prediction scores is that the mutation has occurred in isolation while other factors remain normal, although the reality is not that simple (e.g. other nearby mutations that modify the secondary structure of DNA may counteract the impact of the mutation of interest). It is challenging to investigate such multiple anomalies in a general way because they often occur case by case. To avoid over-interpretation of the prediction score for a single mutation, one should always put it into the context of its local environment (including *cis*- and *trans*-acting splicing elements) and make judgments comprehensively. As always, the predicted findings should be validated in a wet lab, if possible.

## CONCLUSIONS

In summary, our results support previous findings that PWM and MES have the best performance in predicting splice-altering scSNVs. We constructed new models that further improved prediction accuracy. Application of our models to the COSMIC dataset indicated the importance of splice-altering scSNVs in cancer pathogenesis. The present study also provided a resource of directly interpretable prediction scores with very high accuracy for all potential scSNVs across the human genome, which will significantly facilitate splicing defect prediction and detection in both basic and clinical research, thus contributing to the discovery of new targets for gene therapy and newborn screening.

## SUPPLEMENTARY DATA

Supplementary Data are available at NAR Online.

SUPPLEMENTARY DATA
