# Supplementary material for: In silico prediction of splice-altering single nucleotide variants in the human genome
Source: Nucleic Acids Res. 2014 Nov 21;42(22):13534–44. doi: 10.1093/nar/gku1206 (PMC4267638; doi:10.1093/nar/gku1206)
Supplement: SUPPLEMENTARY DATA [file supp_42_22_13534__index.html]

 In silico prediction of splice-altering single nucleotide variants in the human genome — In silico prediction of splice-altering single nucleotide variants in the human genome — SUPPLEMENTARY DATA 

# *In silico* prediction of splice-altering single nucleotide variants in the human genome

## SUPPLEMENTARY DATA

**Files in this Data Supplement:**

- SUPPLEMENTARY DATA
